# Extracts from the Proceedings of the Section on Stomatology of the American Medical Association

**Published:** 1904-07-15

**Authors:** 


THE
### DENTAL REGISTER.

Vol. LVIII. JULY 15, 1904. No. 7.

### EXTRACTS FROM THE PROCEEDINGS OF THE SECTION ON STOMATOLOGY OF THE AMERICAN MEDICAL ASSOCIATION.

(As published in the "Journal").

SYMMETRY IN DENTAL EDUCATION: Dr. G. F. Eames, of Boston, makes a strong and sensible plea for a more systematic and logical education of the dentist, not only from the technical and professional aspect, but preparatory, and in that gradual and completed education which results from the practice of the profession. He deprecates concentration, and advises the cultivation of allied sciences and arts; even literature and civic and economic problems have a cultural value that broaden one's sympathies and make for better professional services.

DEFECTS IN OUR PROFESSIONAL LIFE: Many graduates of professional schools are like statues, carved to be placed in a frieze, the side presented to the public gaze defies criticism, but the other side is of rough, unhewn stone.

PRELIMINARY TRAINING: An ideal course of training should fitly train, not only the mind, but the heart and the body. A record of tendencies as expressed in a child's actions should be kept as a means of determining possible avenues of successful vocations. A child's training should include every branch of activity, pursuing none to excess, and each to a sufficient extent to determine possibilities. Such training and records would make it easy to determine the calling in which the youth would most likely succeed.

Training for Dental Education: Graduation from a high school may be used as a *basis* for admission to the dental college, but it is not always a sufficient standard. Many high-school graduates are utterly unfit to take up the study of dentistry and will never successfully practice it. On the other hand, some men do not need a high-school education to prepare them to enter a dental college, and some do not even need a four-year course to prepare them for practice. There will be varying abilities from one who will never become skillful to the occasional man who will master his professional training in one half the accustomed time. They will attain high places in spite of entrance examinations or college curricula, and they are so well endowed mentally and physically that they are able to decide as to their own fitness for a professional career. Quality, not quantity is requisite, and were it not for the matter of administration difficulties the time to be spent in college would not be a matter of significance. Blunthead would be unfit after a ten-year's course, while Sharp would measure up to the average graduate in two years. The end of the college course is really but the beginning of studentship, at any rate. The graduate takes control of his education and makes it what he will, as modified by his environments, and it devolves upon him to make it as symmetrically as may be, and the wise man will make his life what it should be, while the dullard will drift into a rut or a life of inconsequence.

Specialists: Some practioners develop their mechanical powers and become so absorbed in these specialties that they can see nothing but operative or prosthetic restorations after their particular ideals. Others see nothing so distinctly as commercial success, and others some fad pursued for a little to be replaced by a new one. Specialists have done much to advance particular fields of work, and should be commended for demonstrating possibilities by attention to details and consecration to a single purpose. But usually such efforts carry neglect of other associated matters of vital consequence and lead to a symmetry in development of character. Other attributes of our characters become atrophied through neglect. We should maintain a broad grasp of the whole field while concentrating our effort on any particular part.

EVOLUTION IN EDUCATIONAL STANDARDS: Dr. John S. Marshall, of San Francisco, contributed a brief resume of the development of our present educational standards, beginning with the organization of the first dental college in 1840, when the entrance requirements were, "a desire to learn dental practice" and the curriculum embraced only the fundamentals of anatomy, physiology, chemistry and the principles of operative and mechanical dentistry by the laboratory and chair methods of instruction. This was followed by the organization of other colleges, dental societies, text-books, journals and the present conditions of improved schools and new ideals as to methods of instruction and practice, under State control and supervision, until what was at one time considered to be merely a specialty of medicine has come to be one of the recognized professions.

EDUCATIONAL DEMANDS OF TO-DAY: The great need for the future progress of the profession is for a better fundamental training, which can, in Dr. Marshall's opinion, be best secured by a more thorough preliminary training and better instruction in those fundamental sciences upon which the art principles of dental practice must be constructed. The medical sciences have so broadened that a comprehensive knowledge can not be had in the former brief courses, and they are becoming so profound that a mind not properly disciplined to study can not comprehend them. There is a great demand for science teachers who know their subjects and have capacity of mind and heart which will enable them to impart instruction. In many colleges fairly good equipment is available, while in some there is a great lack. There is great need for the elimination of the commercial aspect in dental education. Combination courses in liberal arts and professional sciences are being organized in many colleges and universities, which, if extended to the dental departments, would lead to a better educated profession. Such men have usually been taught social and political ethics, and they would naturally fall into sympathetic relations with our code of professional ethics.

ADVANCED STANDING: There is no good reason why students who have received thorough training in the science courses of a literary college, or have had thorough training in the medical sciences of a good medical college, should not be allowed to complete the dental course in less time than the man who has not had similar advantages. Since the better medical schools now require a high-school graduation for admission and four years for graduation, it does seem as though these graduates should be given two years of credit on a four-year dental course.

ATTITUDE OF THE PROFESSION AND EXAMINING BOARDS TOWARD A RETURN TO THE THREE-YEAR COURSE: Dr. C. C. Chittenden, in an address to the section on Stomatology, gave a sketch of the actions taken to establish the four-year course. He concludes it would be a decidedly retrograde step to return to a three-year course, and he intimated that the profession, through the examining boards, would resent such a movement and compel the colleges to adopt higher standards or go out of business.

ENDOWED VERSUS COMMERCIAL DENTAL COLLEGES: Dr. A. E. Baldwin, of Chicago, in a paper before the section on

Stomatology, observes that it is his judgment that the hope of bettering professional education in this country lies in the effort and tendency to support those schools which have a vital connection with and receive their support from universities either endowed or supported by the State. He expects to secure in this way higher class teaching and to eliminate the opportunity for personal gain of a financial kind, which are the two prominent obstructions to progress in the present system of private enterprises.

THE POLICY OF HARVARD DENTAL SCHOOL: President Elliot contributes the following statement outlining the reason for the action taken by Harvard Dental School in withdrawing from the Faculty Association. The policy of the Harvard School is not commercial in the least. It is steadily endeavoring to raise the standard of dental education, and has in a good degree succeeded. Just now it is trying to raise the standard of its admission examinations, and is finding that a difficult task. It can not therefore add to the length of its course. Moreover, the school has been struggling for several years to maintain a close connection with Harvard's medical school, which task has proved difficult because of the rapid improvement of the medical school, consequent on its admitting none except such as hold a degree in arts or sciences. The dental school must accomplish both of these tasks before it can extend its course to four years. The section on Stomatology may rest assured that the Harvard Dental School will continue to do what it always has been doing—namely, contribute to the preparatory course for the dental profession.

HARVARD'S SITUATION: Prof. E. C. Briggs wished to emphasize the fact that it was not the intention of Harvard to stand in the way of any advance in educational standards; on the contrary, it was the desire of the faculty to make substantial advances as fast as possible. Harvard, because of its situation, was compelled to make these advances in a different way from that suggested by the National Association of Faculties. We have decimated our classes several times, because of advances made beyond the requirements of other schools and the standards of the National Association of Faculties. It is a question that has not yet been answered, as to what constitutes advance and the proper method of reaching it. Harvard believes it lies in giving its students broader scholarship, and we hope to accomplish this by increasing the entrance requirements. If we increase our entrance requirements to the extent we plan and carry a four-year course, discrepancy between our standard and that of the Faculties Association would be so great that it would practically exterminate our school. It is not, however, commercialism that dictates our action; we teach now in three years as much time as the four-year course requires with one month's exception, and our entrance requirements and fees are higher than the standards of the Faculties Association, so we think we should not be charged with commercialism in this matter. We do not accept students from other schools unless they can pass examinations on all the subjects on which they enter with credits. The Harvard entrance examinations are considerably more exacting than any high-school credits. We are convinced that the men who enter the dental profession must be men of education and scholarly attainment, and believe our method of preparation is the true one. The time will come when Harvard will require four years; she will also demand other things that mean advancement. Harvard's faculty concluded both steps could not be taken at this time and decided to take the one that seemed most urgent.

Harvard's Action an Encouragement to Commercial Schools: Dr. James Truman said he did not want to appear as an antagonist of Harvard Dental School, but he fully believed the action of this school in not going on with a four-year course was responsible for the present agitation in favor of returning to a three-year course. This is the unfortunate consequence of an action that he and no other sane man would say was based upon a selfish idea. The example of Harvard gave support to such schools as know that they can not maintain four courses of instruction that will profitably occupy the student's time. All true educators believe in increasing educational standards as fast as they can be done with safety. The standards vary, or what is more worthy of consideration, methods vary in the minds of every teacher. The idea that because a man has an academic degree, he will make a better dentist than the man who comes with a high-school training, in which he has had a considerable amount of mechanical training, is absurd to me. There is so much that is technical in our calling, that the earlier a man begins to learn to do things with his fingers the better dentist he will make. I fear that we shall make a grave error in putting off the technical training until the last two years of the college course, and admitting students to the course only after they shall have earned an academic degree. The absurdity of the plan may be clearly seen when we remember that from four to six years are required to educate an ordinary mechanic, while this proposition to educate a man to practice the most intricate profession in existence is to be done in less than eighteen months and at a period when the student can not acquire as easily as at an early age.

THE NECESSITY FOR A FOUR-YEAR COURSE IN UNIVERSITY SCHOOLS: Dr. N. S. Hoff, of the University of Michigan, gave the reasons for the adoption of the four-year course by that school. The situation at Michigan is quite similar to that at Harvard. The medical science subjects of the curriculum are taken from the same instructors and in the same classes with the medical students. Owing to the wonderful development of these subjects in the past few years, they have continually broadened, so that they not only take more time, but they require educated men to successfully comprehend them. This necessitates the raising of our entrance requirements to a high-school graduation, and because of the encroachment of the science studies on the time of the technical subjects, the extension of the course to four years. We met the situation by doing both at the same time, and two years in advance of any similar action by other schools. As was expected, a very considerable decrease in attendance followed; but we are pleased to report that we believe a substantial advantage has come in the character of students secured and we hope in the product we shall turn out. There seems to be a generally accepted notion among pedagogic thinkers that where technical subjects are to be taught, that the students should not only be prepared intellectually and sympathetically, but that these should be begun at the earliest practicable opportunity. Under our present system there seems to be no way of beginning a dental education which necessitates a mastery of the fundamental medical sciences much before a student has graduated from the high school. The introduction of manual training in our secondary schools will be a wonderful help in the future. It does not seem that seventeen years, which is the usual age of high-school graduates, can be too mature an age for the successful acquirement of the mechanical technique required in the practice of dentistry. A properly adjusted system of technics, carried systematically through the first three years of a four-year course of instruction, ought to produce results that will be satisfactory. This arrangement of the work would necessarily throw the science and clinical work toward the end of the course. And this would have its advantages, especially one that is important—it would enable the student to adapt the scientific facts that he learns to his clinical experience, and consequently take away the perfunctory disposition he too often maintains toward these subjects. Incidentally he would also be better prepared to take State Board examinations.

The attempt to grade all schools on the same basis is an artificial substitute for the real estimate, which, though often biased and sentimental, exists in the profession and all who take notice of the methods adopted in our various schools. The students themselves very soon estimate and rate the colleges accordingly to their ideals of work accomplished. Therefore, so far as results are concerned, it would be much better if we had no legislation on this subject. Discussion of course we must have, to formulate ideals and stimulate progress, then a laudable emulation would soon prove that those who lead in real worth would be best considered.

AN IDEAL IN DENTAL EDUCATION: Dr. E. A. Bogue, of New York, cited the case of the education of his son, who first was trained by himself in his own office and then went to a dental college and graduated. He then studied medicine and knew what he wanted to get from the medical course. He is now prepared to practice dentistry in all its phases It is this kind of an education that it seemed to him we need in this day when the dentist is called upon to do all kinds of operations in the mouth from extracting a tooth to setting broken jaws and cleft palate operations. The office pupilage method is somewhat antiquated, but when properly conducted it has great possibilities.

DEFECTS IN EDUCATION: Dr. M. L. Rhein, of New York, suggests that the greatest drawback to adopting the high-school graduation as the standard of admission to dental college lies in the fact that this education has little to commend it in the way of preparation for such an exacting course of study as is required for dentistry. The suggestion of introducing the technics of dentistry first in the course will not suffice. The introduction of manual training in our primary and secondary schools will do much to prepare students for taking up professional studies which require so much technical work. It is doubtful if colleges can hope to turn out in every graduate a thoroughly competent dentist. Much that qualifies is inborn, and it is impossible to train others to even a medium degree of skillfulness.

This is a point that some schools notice, and they decline to carry men of recognized incapacity; but the commercial school will continue to graduate any such as they come to the end of the course regardless of qualification.

MEDICAL INSTRUCTORS OF DENTAL STUDENTS: It is a well-recognized fact that medical science teachers who have no knowledge of dental practice make bad work trying to educate dentists. Some method of overcoming the present conditions will have to take place soon or we shall deserve the ridicule the medical profession now throws at us on every occasion. We must have not only time enough, but teachers of the proper sort to educate dentists on the same plane that medical practitioners are now being educated.

DENTAL EDUCATION SHOULD BEGIN EARLY: Dr. E. C. Kirk, of Philadelphia. The lack of a properly-trained mind is due to the faulty training in the formation period of mental development. The kindergarten school is the place to begin preparing the dentist. A mind that has no power of election can not be taught election. The study of mathematics has certain values to those whose business it becomes to count, etc., but the mental discipline a child gets from mathematics in precision is the important factor. This is one of the difficult things dental students have to learn, possibly because so much of our work is not upon a mathematical basis. But as an element of character it is an invaluable asset, because it helps him to be logical. The preparatory training of dental students is of as much significance almost as the college curriculum. If it were practicable, the manual training should be specified. I don't think the old apprentice system can be substituted for the present well-organized technical courses, and think the medical course should be superadded rather than taken first. I believe it is not practicable to produce an ideal dentist from the raw material in less than four years.

